# Fucosterol exhibits selective antitumor anticancer activity against HeLa human cervical cell line by inducing mitochondrial mediated apoptosis, cell cycle migration inhibition and downregulation of m-TOR/PI3K/Akt signalling pathway

**DOI:** 10.3892/ol.2022.13618

**Published:** 2022-12-01

**Authors:** Hongye Jiang, Jie Li, Aiyue Chen, Yinguang Li, Meng Xia, Peng Guo, Shuzhong Yao, Shuqin Chen

Oncol Lett 15: 3458–3463, 2018; DOI: 10.3892/ol.2018.7769

Following the publication of this paper, it was drawn to the Editors’ attention by a concerned reader that the cell migration assay images shown in Fig. 5 were strikingly similar to data appearing in different form in other articles written by different authors in different research institutions. Owing to the fact that the contentious data in the above article had already been published elsewhere prior to its submission to *Oncology Letters*, the Editor has decided that this paper should be retracted from the Journal. The authors were asked for an explanation to account for these concerns, but the Editorial Office did not receive a satisfactory reply. The Editor apologizes to the readership for any inconvenience caused.

